# Concussion in community Australian football – epidemiological monitoring of the causes and immediate impact on play

**DOI:** 10.1186/s40621-015-0052-5

**Published:** 2015-09-10

**Authors:** Lauren V. Fortington, Dara M. Twomey, Caroline F. Finch

**Affiliations:** 1 Australian Centre for Research into Injury in Sport and its Prevention (ACRISP), Federation University Australia, SMB Campus, PO Box 663, Ballarat, VIC 3353 Australia; 2 Faculty of Health, Federation University Australia, Ballarat, Australia

**Keywords:** Sports medicine, Concussion, Head injury, Injury prevention, Injury epidemiology, Return-to-play protocols

## Abstract

**Background:**

Head injuries, particularly concussion, are a major cause of concern in many sports, particularly the football codes, driving a need to better understand injury mechanisms and potential methods of prevention. The aim of this study was to describe the mechanisms and follow up care of concussion injuries sustained in adult male community Australian football to identify target areas for prevention and management.

**Methods:**

Secondary analysis of injury data collected in a cluster randomised controlled trial in community Australian football across two states of Australia in 2007 and 2008. There were 1564 players from 18 clubs. The main outcome measures were the number and rate of head/neck/face (HNF) injuries and concussion sustained in games. A specific description of the mechanisms of the concussion injuries is presented along with the immediate return-to-play status of concussion cases.

**Results:**

143 HNF injuries were sustained by 132 players. The game HNF injury incidence was 4.9 per 1000 game hours (*n* = 138; 95 % confidence interval 4.1; 5.7). Just under a quarter (*n* = 34) of all HNF injuries were recorded as concussion. All concussions occurred during games (none in training), with all but one related to body contact with other players. Overall, 68 % of the concussions were considered within game rules, while 32 % were either outside of the rules or unclear. Most (88 %) players left the field immediately following concussion but 47 % later returned to play in the same game.

**Conclusions:**

Prevention strategies for concussion need to be based on knowledge of the mechanisms of injury. Most concussions in community Australian football occurred through body contact with other players or during tackling. Management of players post-concussion was generally poor with over half of the cases continuing to play in the same game. Therefore, new primary prevention strategies that target body-contact/tackling skills and improved secondary prevention measures relating to compliance with return-to-play protocols would be valuable.

## Background

The risk of head, neck and face (HNF) injuries is an ongoing issue in many sports. Of these, concussion is of particular concern due to the potentially serious consequences that can arise from damage to the brain. Several studies have suggested that the number of concussions reported in sport has increased in recent years (Finch et al. 2013a; Marar et al. 2012) and efforts to prevent and manage concussion have grown accordingly. This is particularly evident in the various forms of football where concerns of players, parents and sporting bodies, have driven the increased clinical and research focus on concussion (Harrison 2014).

Each high profile case of concussion from any of the football codes (including Association football/soccer, rugby league, rugby union, American football and Australian football) motivates new calls in the popular media to mandate headgear use, particularly in community and junior competitions. These views assume firstly, that appropriate helmets are available for use in the sport to ameliorate any impact mechanisms of injury and, secondly, that there are no other possible preventive solutions that could be adopted. For the common football codes played in Australia, the current scientific evidence does not support any protective effect for concussion from many of the soft-shell helmets available (McIntosh et al. 2009; McIntosh and McCrory 2001). Given the lack of effectiveness of headgear in protecting against concussion, other potential preventive solutions need to be considered.

Fundamental principles behind the identification and design of appropriate injury control strategies stipulate that strategies must be based on understanding the mechanisms (energy exchange) behind injury causation (Haddon 1973; McClure et al. 2004). Research into concussion in the football codes to date has largely focused on two themes: the first being helmets for prevention and the second being concussion management. In reference to Haddon’s ten injury countermeasures, these strategies relate to levels six (separation by a material barrier) and nine (rapidly detect and evaluate damage, minimise negative consequences), respectively (Haddon 1973). Strategies that are aimed at avoiding situations leading to concussion occurring in the first place (i.e. levels one through seven) have had substantially less attention in recent research. There is some information about the mechanism of head injury at the elite level of soccer and rugby league where concussion occurs most commonly as a result of impact and collisions or through incidental body contact when tackling (Andersen et al. 2004; Hinton-Bayre et al. 2004). However, similar details for community level football players and all football codes are needed before specific preventive solutions can be recommended for that setting.

Apart from helmets, the second theme of much concussion research across the various football codes has focused on post-concussion management, including immediate treatment of injured players and protocols to govern their return to play. International guidelines for concussion management in sport have been in place since 2001 (Aubry et al. 2002). These guidelines have been updated regularly by leading research and medical specialists and endorsed by peak football bodies across all codes (McCrory et al. 2013). Despite these endorsements, the degree of implementation of the guidelines and adherence to the recommendations, at all levels of the sport, has been questioned. Of greatest concern is the apparent lack of implementation of return-to-play protocols (Hollis et al. 2012; Haran et al. 2015).

This study aimed to address some of the limitations above in our understanding of concussion in the setting of community Australian football, through a secondary analysis of epidemiological data reported in a group clustered randomised controlled trial (cRCT). Specifically, data is presented on the circumstances in which concussions occurred in community Australian football players and the immediate within-game return-to-play status of the concussed players.

## Methods

### Procedures

A secondary analysis of prospectively collected injury data from the Preventing Australian Football Injuries through Exercise (PAFIX) cRCT was undertaken (Finch et al. 2009). Full details of the PAFIX study design and data collection procedures have been published (Finch et al. 2014b; Twomey et al. 2011; Finch et al. 2009) with a summary of relevant aspects provided in this paper. Information about the accuracy of the injury data collection procedures and the assigning of OSICS codes to all provisional injury diagnoses have also been published (Finch et al. 2014a; Twomey et al. 2011). All resources are available online from pafixproject.wordpress.com.

A primary data collector (PDC), who was a sports/exercise science student and/or sports trainer, was allocated to each team participating in the PAFIX trial and attended a formal education session about injury recording for this trial. Medical staff associated with the club assisted with checking the injury data recorded by the PDCs. The PDCs were provided with standardized data collection forms to record both game/training attendance (hours of participation) and details of any injuries sustained. These forms were completed each week for training and games and returned to the research team for processing.

### Outcomes

An injury was defined as “something that caused a player to seek medical attention (on or off the field) or to leave the field of play.” Injury details recorded by the PDC from observation and substantiated by the player and medical staff included: the nature of injury, body region injured, likely injury mechanism, a text narrative description of the injury-causing event and whether or not the player immediately left the field/training area for treatment/assessment and if they returned to the same day. Concussion was diagnosed by medical staff in charge of the team based on concussion symptoms experienced by the player at the time of injury. The PDCs recording the injuries also attended every training session so most diagnoses were followed up with players and confirmed at subsequent training sessions. Based on this reported detail, injuries were subsequently coded according to the Orchard Sports Injury Classification Scheme version 10 (OSICS-10) (Finch et al. 2014a).

Information about the mechanism of injury was extracted from both the pre-coded injury mechanism data field on the injury surveillance form (which categorised the injury as occurring as part of a tackle, a collision, being struck/hit, or other) as well as from the narrative text description of the injury event. Details on whether the injury event was considered within the game rules or not was based on both whether the official signaled a foul and from the players narrative recorded on the injury form which captured off-the-ball (behind main play) events. Information about the same day actions in relation to leaving the field for treatment/assessment and then returning-to-play was also taken from the injury reporting form.

### Subjects

Overall, 1564 male community (amateur) Australian football players, aged ≥18 years, were followed up over one playing season in either 2007 or 2008. Players came from 18 clubs across two states, in regional and metropolitan areas. A season consisted of 26 weeks (pre-season and regular season) and clubs typically held 2 training sessions per week, with one competitive match scheduled on weekends.

### Analysis

For this sub-study, data were extracted on all players who reportedly sustained a HNF injury during the study period (i.e. if the first letter of the OSICS-10 assigned to the injury was H.) Descriptive epidemiological data on the mechanism of injury and return to the field of play are presented along with the injury incidence rate, calculated as the ratio of the number of head injuries sustained to hours played (separately for games and training, as well as combined), with 95 % confidence intervals (95 % CI). Analyses for this study were performed using Microsoft Excel 2013, IBM SPSS Statistics version 21 and OpenEpi.com.

### Ethical considerations

The PAFIX study received ethical approval from the University of Ballarat (now Federation University Australia) and the University of Western Australia Human Research Ethics Committees.

## Results

### Injury rates

Of 1032 injuries sustained by the 1564 players, 143 injuries (14 % of all injuries) were to the head, neck or face (HNF). Of these, 53 % (*n* = 75) were to the head/neck, 39 % (*n* = 56) were to the face; and 8 % were to the mouth/teeth (*n* = 12). Most (97 %) of these 143 injuries were sustained during competition games, with five injuries occurring during training. The HNF injury incidence rate overall (training and games combined) was 2.1 per 1000 participation-hours (95 % CI: 1.8; 2.5). Focussing on games only, the HNF injury incidence rate was 4.9 per 1000 game hours (95 % CI: 4.1; 5.7).

Of the 75 injuries to the head/neck, fewer than half were classified as concussion (45 %, *n* = 34), equating to an overall frequency of concussion of 3.3 % of all injuries. The concussion injury incidence rate overall (games and training combined) was 0.5 per 1000 participation-hours (95 % CI: 0.4; 0.7) and in games only, it was 1.2 per 1000 game hours (95 % CI: 0.8; 1.7). All cases (100 %) of concussion occurred during games (none in training.)

### Mechanisms of concussion

Detailed information on the circumstances associated with the 34 cases of concussion is presented in Table 1. The majority (68 %) of concussions were reported by the PDCs as having occurred within the game rules. The descriptive case narratives of the injury event indicated that almost all concussion cases were sustained through direct player-to-player contact. One case occurred when a player landed poorly and hit his head when attempting to jump and mark (catch) the ball.

**Table 1:** Circumstances surrounding the incidents resulting in concussion in community Australian football players (*n* = 34)

Cause	Number	Percent	Description of injury by case
Collision with another player/umpire^a^	13	38	Hit on the head while contesting the ball.
			Player had their head over the ball and opposition player came through and knocked him out.
			Slipped in possession and got a knee in the head, blurred vision.
			Hit his head on ground after being knocked by opposition player.
			Back of neck. Ran into player who pushed head down.
			Went up in pack - got crunched.
			During the collision with another player, player sustained a suspected torn quad muscle. Was treated for light concussion when he hit his head.
			Shoulder hit head and head flung back.
			Stuck between two players and got hit in the head.
			No further details given.
			No further details given.
			No further details given.
			No further details given.
Involved in tackle	13	38	Kneed in the head during a tackle, very disorientated.
			Banged heads in tackle.
			Got tackled high and felt dizzy.
			Sustained an elbow to the face, resulting in a blood nose. Player later complained of blurred vision, dizziness, and headache and felt sick in the stomach. Report during the next week confirmed player has fractured nose and cheekbone, as well as mild concussion.
			Got slung to the ground and hit his head on the hard ground.
			Player sustained a heavy blow to the head during a tackle.
			Was going for the ball and was hip & shouldered then tackled. Split bottom lip and concussion.
			Got tackled and his arms were pinned to his sides, landed on his side and head hit the ground.
			Opposition player landed on his head.
			Landed on hard ground in wicket area^c^
			Clashed heads with another player.
			Got his head pushed into the ground when he was tackled.
			No further details given.
Struck or hit^b^	7	21	Struck on head while going for a mark.
			At ground level charged by opposition & knocked in head. Got up straight away but taken off as precaution.
			Hit in the head in an off the ball incident.
			Received a knock on the head during a mark and then landed with head hitting the ground.
			Got elbowed in the head very hard.
			Hit by opponent in an off the ball incident. Minor concussion and was observed for a few minutes, then went back on.
			No further details given.
Other	1	3	Fell back on his head while landing from a mark.

^a^collision injuries involved the injured player impacting with another

^b^hit/struck injuries were initiated by another player (injured player was passive in the action)

^c^Wicket area refers to a hard surface in the middle of the ground that is used for the game of cricket

### Return to play behaviours

Investigation of the immediate actions of players following the game-related concussion incidents showed that most players (88 % of the concussed players) left the field of play immediately after the incident. Four players continued to play despite an injury report suggestive of a concussion. Fourteen of the 30 concussed players who left the field (47 %) later returned to exactly the same game; all had been attended to by a sports trainer or physiotherapist at the ground.

## Discussion

This study is the first to provide detailed information about injury mechanisms associated with concussion in community Australian football. While there is some understanding of the specific causes of similar injuries at the elite level in other forms of football (soccer, rugby league) (Andersen et al. 2004; Hinton-Bayre et al. 2004) information surrounding concussion events in community sport is very limited. This has resulted in a general lack of measures that are aimed directly at preventing the occurrence of situations that lead to concussion. Much of the recent literature around concussion has limited its focus to the adoption of management guidelines post-concussion or to the effectiveness of headgear, rather than primary prevention (Hollis et al. 2012; Feddermann-Demont et al. 2014). Although important, such approaches correspond only to the later-stage levels of countermeasure strategies, outlined by Haddon (Haddon 1973). Implementing return-to-play protocols is essential for protecting against adverse health outcomes of concussed players, however, they do nothing about preventing the injuries from occurring in the first place. Continued investigation of potential primary prevention measures is needed in addition to the ongoing monitoring of return to play practices (Johnson 2012). Better understanding of the mechanisms of injury is critical for this.

In this study, all concussions sustained occurred during games (not in training), and, all but one, were the result of direct contact between players or during tackling. The new insights gained on when these injuries during games should be useful for targeting potential preventive strategies during training sessions. For example, future research could look into the effectiveness of training exercises for body-contact, neck strengthening measures, safe tackling approaches, etc. Additionally, in this study, at least 18 % of concussions, and as many as 33 % (inclusive of those that were unclear), were attributed to play that was considered to be outside of game rules. While we have no knowledge of the specific enforcement of rules surrounding these incidents, educating players and coaches on why these rules exist and reviewing the enforcement of games rules could be valuable strategies to reduce dangerous play that leads to injury. Our results support further inquiry as to when, why and how concussion injuries occur to elucidate these potential alternative preventative measures.

In comparison to other codes of football, the proportion of players who sustained a concussion was relatively low in this group of community Australian football players. Overall, HNF injuries collectively accounted for 14 % of all injuries over two seasons of community Australian football; with less than a quarter of these being concussions, just 3 % of all injuries sustained. This proportion is consistent with previous studies in the adult community Australian football setting (Braham et al. 2004a; Braham et al. 2004b; Braham and Finch 2004; Finch et al. 2005). While concern about the seriousness of concussion is certainly reasonable, popular fears about a high rate of Australian football players sustaining this injury are likely to be over-estimated.

Although helmets have received the majority of attention as a method for reducing the occurrence of concussion, alternative measures also need consideration. This is demonstrated by the range of injury incident scenarios shown in our study. A recent systematic review of potential modifiable factors for preventing concussion highlighted the range of possible areas for consideration, including rule changes, equipment (in addition to helmets, e.g. mouthguards) and neck strength (Benson et al. 2013). However, only a handful of studies were identified for inclusion and there was no strong evidence arising from these studies in favour of any particular strategy (Benson et al. 2013).

While primary prevention measures should be pursued, the potential severity of concussion outcomes means research on the implementation of best practice management will not be, and should not be, disregarded. Despite the long history of guidance on how to manage concussion in sport, many cases are still not handled according to the recommended return-to-play protocols (Hollis et al. 2012; Haran et al. 2015). Although a positive finding was that most players initially left the field, ensuring compliance with return to play guidelines is important. A substantial number of players in this study later returned to play in the same game in which the concussion had occurred. This is particularly concerning in respect of their injury descriptions being indicative of the signs and symptoms of having sustained concussion, and indeed that being the diagnosis given to their injury.

A team sports trainer or physiotherapist reportedly attended to all the players who later returned to the field. The actual assessment of players or advice provided is not known and it may be that players returned to the field on their own accord, against the instruction of coaching, medical and first aid staff. It should be noted that both player and sports trainer behaviour and knowledge may have changed since data were collected for this study in 2007-2008. However, even at that time, the first consensus guidelines had already long stated that if a player was showing any signs or symptoms of concussion they should not be allowed to return to play in the current game/training (Aubry et al. 2002). In light of our results and with recent evidence still finding that knowledge of return to play guidelines is largely inadequate (Newton et al. 2014; White et al. 2014a) continued education on concussion protocols for both players and support staff is warranted. This has been identified as a major challenge (White et al. 2014b; Provvidenza et al. 2013; Finch et al. 2013b) but certainly, one that is necessary to address. In 2013, the Australian Football League (the peak body for Australian football) produced and disseminated new concussion guidelines on their community website and these are promoted as best practice for the game. More research will be needed to show whether people are aware of these new guidelines for concussion in community Australian football and if players and clubs adhere to them.

The strength in the data collection procedures for the PAFIX trial mean that it is unlikely that injuries were unreported. The epidemiological field-based data collection procedures were found to be highly reliable (Twomey et al. 2011) and were based on those used by the authorship team in other similar sports epidemiology and injury research (Braham et al. 2004a; Finch et al. 2005). As there was no medically-qualified person involved in the reporting of the injuries, it is possible that some players were misreported as either having or not having sustained a concussion. Nonetheless, the text narrative descriptions of the circumstances around the incidents associated with the injuries coded as concussion are consistent with this injury. Concussion can be a hidden injury and players may have tried to negate any potential diagnosis. This non-reporting has been described in a recent study with players not thinking the symptoms were serious enough to report or fear of being removed from participating (Delaney et al. 2015). Based on this, the results from our study might underestimate the concussion incidence. However, the data collection reliability assessment (Twomey et al. 2011) and having the PDC’s embedded in the team training and games, suggests that the chance of having missed injuries is very low. Although sport concussion management guidelines have changed since these data were collected, return to play protocols at the time presented a similar message on return to play as current (Hollis et al. 2012). Importantly, the key data describing the mechanism of injuries in this study will not have been impacted by the timing of data collection.

## Conclusions

In this study of over 1500 community Australian football players a relatively low incidence rate of concussion injuries is reported. The detailed injury information provides insight on the events surrounding the concussion that will be useful for injury prevention and management. The majority of concussion injuries resulted from player-to-player contact, within the game rules. Due to the potential for serious outcomes and in light of poor adherence with return to play guidelines, additional research into the most effective prevention measures to address these injury mechanisms, as well as looking further at methods for improving compliance with return to play guidelines for concussion are recommended.
